# Drivers of Bacterial Maintenance and Minimal Energy Requirements

**DOI:** 10.3389/fmicb.2017.00031

**Published:** 2017-01-31

**Authors:** Christopher P. Kempes, Peter M. van Bodegom, David Wolpert, Eric Libby, Jan Amend, Tori Hoehler

**Affiliations:** ^1^Santa Fe InstituteSanta Fe, NM, USA; ^2^Institute of Environmental Sciences, Leiden UniversityLeiden, Netherlands; ^3^Department of Earth Sciences, University of Southern CaliforniaLos Angeles, CA, USA; ^4^Department of Biological Sciences, University of Southern CaliforniaLos Angeles, CA, USA; ^5^NASA Ames Research CenterMoffett Field, CA, USA

**Keywords:** maintenance metabolism, basal power requirement, metabolic ecology

## Abstract

Microbes maintain themselves through a variety of processes. Several of these processes can be reduced or shut down entirely when resource availability declines. In pure culture conditions with ample substrate supply, a relationship between the maximum growth rate and the energy invested in maintenance has been reported widely. However, at the other end of the resources spectrum, bacteria are so extremely limited by energy that no growth occurs and metabolism is constrained to the most essential functions only. These minimum energy requirements have been called the basal power requirement. While seemingly different from each other, both aspects are likely components of a continuum of regulated maintenance processes. Here, we analyze cross-species tradeoffs in cellular physiology over the range of bacterial size and energy expenditure and determine the contributions to maintenance metabolism at each point along the size-energy spectrum. Furthermore, by exploring the simplest bacteria within this framework– which are most affected by maintenance constraints– we uncover which processes become most limiting. For the smallest species, maintenance metabolism converges on total metabolism, where we predict that maintenance is dominated by the repair of proteins. For larger species the relative costs of protein repair decrease and maintenance metabolism is predicted to be dominated by the repair of RNA components. These results provide new insights into which processes are likely to be regulated in environments that are extremely limited by energy.

## 1. Introduction

Understanding the minimal energetic requirements for bacteria has far-reaching importance ranging from estimating total carbon budgets in the deep ocean to understanding the constraints on the origination, survival, and proliferation of life on our own planet and other planetary bodies. However, despite years of research dating to Schulze and Lipe (1964) and Pirt (1965), and a general understanding of maintenance processes, we still do not have an explicit theory for calculating and defining maintenance metabolism *a priori* (van Bodegom, 2007; Hoehler and Jørgensen, 2013; Lever et al., 2015). In order to advance our understanding of maintenance requirements, here we explicitly connect these requirements to cellular processes which may be driving energy investment in maintenance. Different processes have been proposed as the key objective of maintenance metabolism, such as sustaining the proton motive force, osmoregulation, the degradation of macromolecules, and regulated shifts in metabolic pathways (van Bodegom, 2007; Hoehler and Jørgensen, 2013; Lever et al., 2015). Here, we consider systematic trends in cellular physiology that have recently been described empirically and, in some cases, theoretically, for processes spanning the gamut from metabolic rate to protein abundance (Makarieva et al., 2005, 2008; DeLong et al., 2010; Kempes et al., 2012, 2016; Lynch and Marinov, 2015). Our goal is to understand how the detailed processes of a cell, and their differences across species and cell sizes (DeLong, 2010; Kempes et al., 2012, 2016; Lynch and Marinov, 2015), contribute to maintenance metabolism. We specifically consider the energetic costs of protein repair, RNA repair, trans-membrane proton gradients, and motility, each as a function of cell size.

Through our approach, we show that comparing the size dependence of total and maintenance metabolism predicts a lower bound on bacterial size consistent with several recent studies (Kempes et al., 2012, 2016). We also estimate the scaling of the basal power requirement (BPR) across the range of bacterial sizes. We then predict the requirements for maintaining the protein and RNA components of the cell and compare these calculations to the predictions for maintenance and active metabolism. For the smallest bacterial cells with the least amount of metabolic energy, the repair of proteins represents a large fraction of both maintenance and overall metabolism. Our results are consistent with previous analyses of the relative costs of maintaining various components (e.g., Lever et al., 2015) and metatranscriptomic perspectives (Orsi et al., 2016, 2015), but connect these processes to theory and empirical results that describe the cross-species trends in cellular composition and function. These trends highlight different limitations facing bacteria at different sizes, suggest which types of bacteria might be selected in environments with different energy constraints, and, at the smallest end of life, elucidate the limits to cellular function reduction to deal with energy limitation.

## 2. Trends in endogenous, maintenance, and basal metabolism with cell size

The strong interest in energy requirements at slow growth has given rise to a set of distinct definitions which we should be careful to distinguish. Maintenance metabolism, as originally defined (Schulze and Lipe, 1964; Pirt, 1965), represents the metabolic requirements inferred for a zero-growth condition. However, the inference is made from a linear extrapolation measured across cells growing at different rates in steady-state, and thus some of the metabolic context of fast growing cells is carried over to the zero-growth condition. In reality, starving cells make a variety of adjustments to optimize survival under slow growth and this leads to the widely used concept of endogenous metabolism (e.g., van Bodegom, 2007; Makarieva et al., 2008; Hoehler and Jørgensen, 2013; Lever et al., 2015). This metabolism represents consumption rates measured in cells that have undergone some amount of starvation, although the degree of starvation often varies (Makarieva et al., 2008), and, thus the definition of endogenous metabolism is less clear than maintenance where the extrapolation to zero growth is more systematic. A third concept is that of the basal power requirement which represents the true minimum metabolic rate of a cell (van Bodegom, 2007; Hoehler and Jørgensen, 2013; Lever et al., 2015). Here one expects this rate to be both the consequence of regulatory adaptation to low nutrient levels, but also the long-term evolution of cells toward some minimal energy requirement. Thus, *a priori*, from these definitions we expect maintenance metabolic rate to be greater than endogenous rates which in turn should exceed the basal power requirement.

To understand the contributions of different cellular processes to a bacterium's energy budget, we look at cross-species scaling relationships as a function of cell size. This approach has recently been useful in describing cross-species tradeoffs in energetics and physiology, along with bounds for the possible range of sizes for bacteria (DeLong, 2010; Kempes et al., 2012, 2016). We will typically use relationships of the form $Y=Y_{0}V^{\alpha }$ to describe these cross-species trends, where *V* is cell volume, *Y*_0_ is the normalization constant of the scaling relationship, and *Y* is a generic property of interest.

**Figure 1:**
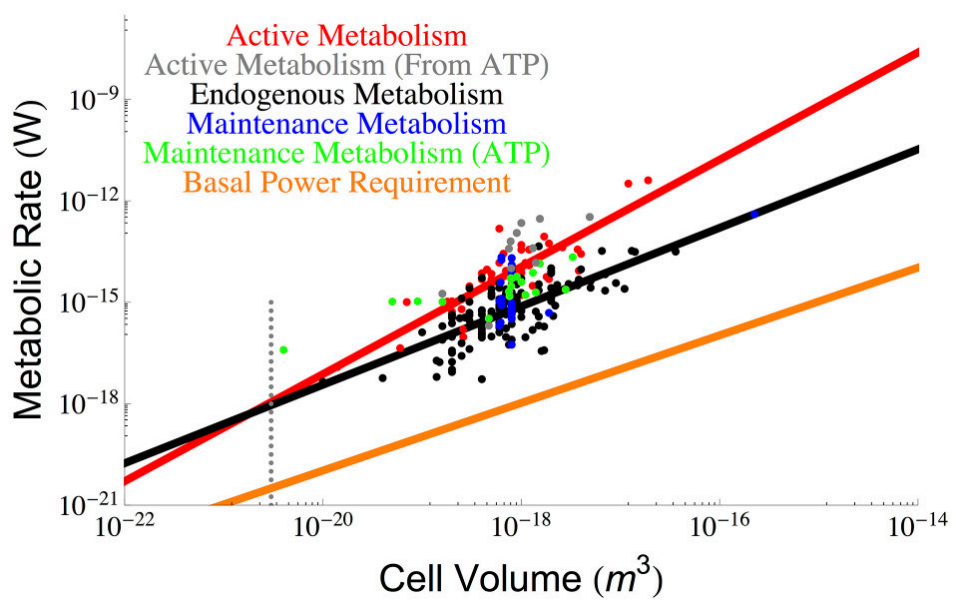
**The overall scaling of total (red) and endogenous (black) metabolism with cell size along with the OLS power law fits**. The data are from the (DeLong, 2010) who reanalyzed two papers by (Makarieva et al., 2008, 2005). Our reanalysis using OLS fits results in a prediction for a lower bound on bacterial size that is consistent with the previous predictions of the cell size at which maintenance is expected to dominate cellular metabolism and reduce the growth rate to zero (Kempes et al., 2012). It can be seen that this occurs at roughly the size of the smallest observed bacteria, and this result adds another limitation to the small end of life along with previously uncovered constraints on growth rate and total space (Knoll et al., 1999; Kempes et al., 2012, 2016). Data for true maintenance rates as defined by Pirt (1965), and estimated from ATP (Lynch and Marinov, 2015) (green points) or glucose and oxygen requirements (Kempes et al., 2012) (blue points) agree on average with the endogenous metabolic rates, although the ATP values are slightly higher. The active metabolic rates estimated from ATP requirements (Lynch and Marinov, 2015), (gray points) are slightly higher than the active metabolic rates reported by DeLong (2010) and Makarieva et al. (2005), which is to be expected because the latter two studies are not exclusively based on values corresponding to maximal growth rates. The dotted gray line indicates the smallest known bacterial cells (Luef et al., 2015; Kempes et al., 2016).

It has been shown that both the active and endogenous metabolism of the cell scales with overall cell size (DeLong, 2010). Using the data from DeLong (2010) we have plotted the ordinary least squares (OLS) fits for the active, *B*_*tot*_, and endogenous, *B*_*m*_ metabolism of the cell in Figure 1, where empirically

(1) $$\begin{array}{l} B_{tot}\propto V^{1.58\pm 0.30} \end{array}$$

(2) $$\begin{array}{l} B_{m}\propto V^{1.16\pm 0.21} \end{array}$$

It is thus apparent that there is a non-constant fraction of endogenous metabolism with overall cell size. In fact, the ratio of total to maintenance metabolism follows the scaling relationship $B_{tot}/B_{m}\propto V_{c}^{0.42}$. More importantly the ratio *B*_*tot*_/*B*_*m*_ will equal 1 at a size of 1.64×10^−21^ m^3^ which should set a lower bound on cell size (endogenous metabolism cannot exceed total metabolism), enforcing a hard constraint on the size of the smallest bacteria. This is only slightly smaller than the lower limit predicted recently by space constraints (4.10 × 10^−21^ m^3^) (Kempes et al., 2016) and is consistent with the smallest observed bacterial cells (3−4 × 10^−21^ m^3^) (Knoll et al., 1999; Seybert et al., 2006; Luef et al., 2015). In addition, it compares well to work that connects metabolic scaling to maintenance and biosynthesis costs to show that growth rate goes to zero at a comparable size of 1.45 × 10^−20^ m^3^, where the maintenance metabolism is also anticipated to become the total metabolism (Kempes et al., 2012, 2016).

Other recent and thought-provoking work has also analyzed the scaling of active metabolic rates, along with maintenance metabolism, in terms of ATP requirements (Lynch and Marinov, 2015). These values can also be used to estimate metabolic rate and are shown in Figure 1. The active metabolic rates are slightly higher than those estimated by DeLong (2010) and Makarieva et al. (2005, 2008). Related to this slight disagreement two features should be noted: first, the values reported in Lynch and Marinov (2015) are rates corresponding to maximum growth, while this is not always the case in DeLong (2010) and the original database from Makarieva et al. (2005, 2008), where endogenous rates were measured from a variety of protocols including cells harvested from different points along the late exponential to stationary growth-phase trajectories (Makarieva et al., 2008); second, the respiration rates from these later studies are direct measurements while many of the ATP values are calculated from other measurements of the cell (Lynch and Marinov, 2015). Given these measurement differences, the scaling of the ATP-related active metabolic rates (Lynch and Marinov, 2015) follows a steeper exponent of 1.92 ± 1.46 (see Section Methods), yet combining these values with Delong et al. still gives an overall scaling of 1.65 ± 0.32. The maintenance metabolic rate estimated from the ATP values are also slightly higher than the endogenous metabolic rates and also follow an approximately linear relationship Lynch and Marinov (2015). However, when we compare the endogenous metabolic rates from Delong et al. and Makarieva et al. to the true maintenance rates estimate from oxygen or glucose consumption in a fourth dataset (Kempes et al., 2012) (blue points of Figure 1), we find that they do not systematically disagree. This suggests that on average endogenous metabolic rates may be a close proxy for maintenance rates given the comparison with similar (e.g., oxygen- and glucose-based) measurements, and accounting for the fact that the ATP energetic estimates generally seem high compared to those calculated from oxygen and glucose consumption. Given this similarity in maintenance and endogenous metabolic rates, we will use these terms mostly interchangeably throughout the rest of this paper, which is a convention used in previous studies (Pirt, 1965) despite the definitional differences highlighted above.

Finally, it has been noted that the minimal energetic requirements for cell survival (basal power requirements or BPR) measured in environments with extreme energy limitation are considerably lower (Hoehler and Jørgensen, 2013; Lever et al., 2015) than the laboratory maintenance values discussed above. Unfortunately, since these measurements have only been made for bulk communities we do not understand how they vary with overall cell size. For comparison with the cross-species scaling relationships described above we can estimate a per volume BPR, assuming a typical cell size and metabolic rate from these environments (Hoehler and Jørgensen, 2013; Lever et al., 2015), and scale this linearly to overall cell volume *B*_*BPR*_ = ζ_*BPR*_*V* (for the value of ζ see Section Methods), which we have done in Figure 1. The BPR trend is about three orders of magnitude below the laboratory maintenance rates as expected from previous estimates (Hoehler and Jørgensen, 2013; Lever et al., 2015). This result again points toward the extreme reduction of cellular function and processes in energy poor environments (Hoehler and Jørgensen, 2013), and provides an important reference for considering the costs associated with various cellular functions as discussed below.

Equations (1) and (2) define the overall expectation for endogenous and total metabolic rates with which we will compare the costs of individual cellular processes across the range of bacterial cell sizes. It should be noted that the relationship between cell size and cellular energetics implies that exploring this range also explores the range of required metabolic requirements. Considering the smallest cells is analogous to considering the lowest metabolic rates and simplest cellular metabolisms which also defines the physiological state of life living under minimal energy requirements (Lever et al., 2015).

## 3. The maintenance cost of individual cellular processes

### 3.1. The cost of protein repair

Drawing on recent work that defines cross-species trends in cellular composition (DeLong, 2010; Kempes et al., 2012, 2016), we consider the maintenance costs associated with various cellular processes. In each case the simplest consideration of maintenance metabolism should be the product of the rate of decay or damage, the expected abundance, and the cost of repairing a unit of damage. For example, the maintenance metabolism, *B*_*p*_ (W), of proteins can be considered as *B*_*p*_ = η_*p*_β_*p*_*N*_*p*_ where η_*p*_ is the specific protein degradation rate (s^−1^), β_*p*_ is the cost of resynthesizing an average protein (J), and *N*_*p*_ is the number of proteins in the cell. This approach of a constant decay rate per protein has successfully been employed in two recent studies to estimate the repair portion of the cellular protein metabolism (Lynch and Marinov, 2015) and the total cellular requirements for ribosomes (Kempes et al., 2016). In both studies it has been shown that $N_{p}=P_{0}V_{c}^{\alpha_{p}}$ where *P*_0_ is a normalization constant, and *V*_*c*_ is the volume of a cell. The relationship for *B*_*p*_ depends on the change in the number of proteins with cell size as defined by the α_*p*_. Empirically it has been shown that α_*p*_ ≈ 0.70 (see Kempes et al., 2016), which disagrees slightly from the value of α_*p*_ ≈ 0.92 found in Lynch and Marinov (2015) (considering only the values for bacteria). However, Kempes et al. (2016) considers 101 data points, and the values from Lynch and Marinov (2015) fall within the range of variation from the larger data set in Kempes et al. The combination of the two data sets does not change the value of α_*p*_ from 0.70, which we employ for calculations here. Our calculations of costs will thus differ from Lynch and Marinov (2015) in terms of the scaling of the number of proteins which has important implications for the smallest bacteria.

From these considerations it follows that *B*_*p*_ increases with increasing cell size according to the relationship

(3) $$\begin{array}{l} B_{p}=\eta_{p}\beta_{p}P_{0}V_{c}^{\alpha_{p}}. \end{array}$$

Since we compare our results to metabolic and maintenance rates that are typically measured at a population level, the population or life-cycle average values for cellular concentrations should be applied here (see Section Methods).

It should be noted that the measured degradation rates include the necessary digestion of proteins as part of the overall regulation and control of cellular function. For example, if a pathway is not needed at a particular point in the cell cycle, then the associated proteins are intentionally eliminated (Maier et al., 2011). Thus, not all of the protein degradation pertains to damaged proteins that must be replaced. For this reason we consider results for the minimum, maximum, and average degradation rates. The minimum observed degradation rates should represent a lower bound on what could be considered the maintenance cost for proteins in the cell as these likely represent the rate of damage alone. It has been reported that protein half-lives in growing *Mycoplasma pneumonia* cells range between 12 and 42 h with a mean of 23 h, with a maximum value of roughly 166 h *in vivo* (Maier et al., 2011). The mean degradation time is then η = 8.37 × 10^−6^ (*s*^−1^) which is similar to other reported values of 3.03 × 10^−5^ (Jayapal et al., 2010) (*s*^−1^) and 2.53 × 10^−5^ (Trötschel et al., 2013; Lynch and Marinov, 2015).

**Figure 2:**
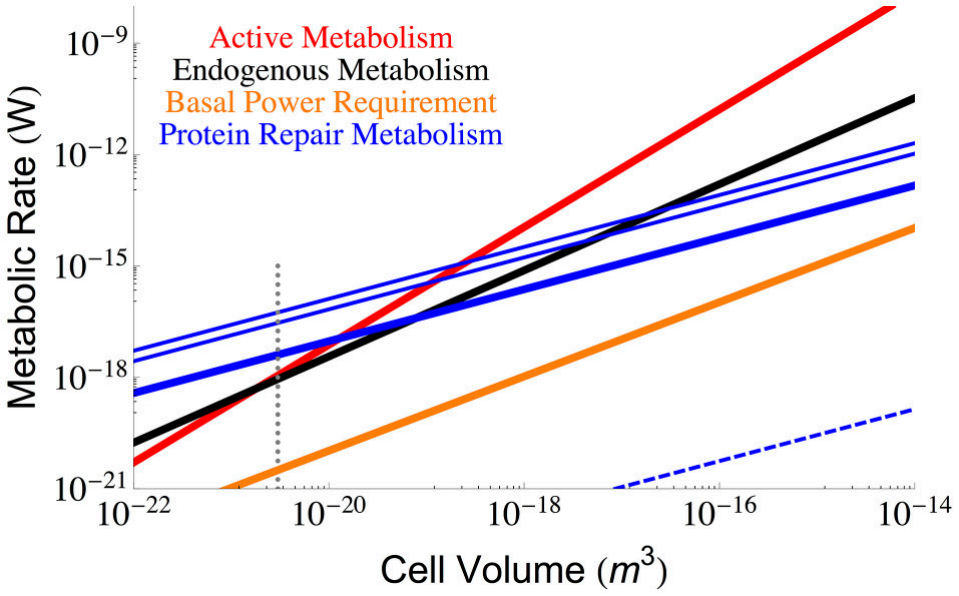
**The overall scaling of total (red) and maintenance (black) metabolism along with predictions for the total protein repair cost given the three protein half-life estimates from growing ***Mycoplasma pneumoniae*** cells (minimum = 12 h, maximum = 166 h, and median = 23 h) (Maier et al., 2011)**. The cost of protein repair becomes the dominant portion of maintenance and cellular metabolism for the smallest cells with the lowest metabolic rates. It should be noted that at these smallest sizes protein numbers may fall below the average trend, and our more detailed analysis of *Mycoplasma* species demonstrates that the protein repair cost would not exceed the total metabolism of the cell. Also note that intentional protein degradation is likely to increase with energy supply (and hence cell size) and thus, the maximum protein repair costs most likely applies to high cell volumes and the minimum repair costs to the lowest cell sizes. The blue dashed line represents the costs associated with the much longer *in vitro* degradation rates of proteins (≈ 20, 000 years Collins and Gernaey-Child, 2001). For each of the protein repair cost curves the reduction of cellular protein abundances in response to energy limitation has not been considered, and this could lower the value of each line by as much as 95% (Lever et al., 2015), although the associated reductions in cell size would also need to be taken into account. The polymerization costs per amino acid (Lever et al., 2015) were used in combination with the average protein length (in amino acids; Kempes et al., 2016) to calculate the cost of protein replacement β_*p*_. The dotted gray line indicates the smallest known bacterial cells (Luef et al., 2015; Kempes et al., 2016).

Using Equation (3) and the measured values of η, β, and α_*p*_ discussed above it is possible to calculate the interspecific protein repair costs of bacteria. Figure 2 shows the metabolic cost of repairing proteins considering the minimum, maximum, and median degradation rates (Maier et al., 2011) compared with the total active and maintenance metabolism of the cell. It can be seen that for the slowest degradation rates the protein maintenance cost falls below the observed maintenance metabolism; however, near the smallest bacteria, the protein cost is expected to dominate and even exceed overall maintenance metabolism (at a size of 7.74 × 10^−21^ μm^3^, which is close to the minimum cell size estimated above and elsewhere Kempes et al., 2012, 2016). Even though protein maintenance is expected to be a dominant component of maintenance metabolism (Lever et al., 2015; Lynch and Marinov, 2015), it might seem concerning that the protein repair metabolism is so close to the total and maintenance metabolism for the smallest cells, especially if we expect other processes to require significant maintenance energy. However, it should be noted that the smallest cells have a reduced protein number implying that the best fit to the data may be more complicated than a power-law with logarithmic curvature at the smallest scales. For example, considering the protein content in several *Mycoplasma* species (Milo, 2013), and using Equation (3), we calculate a maintenance metabolism for proteins of 9.34 × 10^−18^ (W), which does fall below measured total metabolic rate of 2.8 × 10^−17^ (W) (DeLong, 2010). Also, as noted earlier, the maintenance power values from Lynch and Marinov (2015) are larger than those from other sources (Makarieva et al., 2008, 2005; DeLong, 2010), and these would typically exceed the protein repair costs at the small end of bacteria (Figures 1, 2). In general, under more reduced energy conditions the number of proteins could be further decreased to lower repair costs, which is consistent with measurements that show a 95% decrease in protein abundance under starvation conditions (Lever et al., 2015). Similarly, it should be noted that the metatranscriptomes of organisms living under extreme energy limitation are dominated by the expression of protein turnover processes (Orsi et al., 2016, 2015).

Furthermore, it should be noted that the *in vitro* half-life for proteins is approximately 20,000 years (Collins and Gernaey-Child, 2001) (similar rates are reported in Lever et al., 2015), which illustrates that much of protein damage may be a consequence of metabolic activity, and thus repair costs could naturally slow under reduced energy conditions. Taking this much longer half-life into consideration we have also calculated the *in vitro* lower-bound on protein repair in Figure 2, which falls well below the estimate for BPR.

### 3.2. The cost of RNA repair

The cost of maintaining the various RNA pools can be found from *B*_*x*_ = η_*x*_β_*x*_*N*_*x*_, where *x* is the RNA component of interest (e.g., to distinguish mRNA from tRNA), *B*_*x*_ is the maintenance metabolism (W), *N*_*x*_ is the number of a specific pool of RNA molecules (e.g., tRNA, mRNA, or ribosomes), η_*x*_ is the specific degradation rate (s^−1^), and β_*x*_ (J) is the resynthesis cost (the cost to fully cycle a broken RNA component back into the functional form). Previous theoretical work (Kempes et al., 2016) has bounded the number of ribosomes, *N*_*r*_, considering the requirements for both protein and ribosome synthesis, according to

(4) $$\begin{array}{l} N_{r}\geq \frac{{\overset{-}{l}}_{p}N_{p}\left(\frac{\phi }{\mu }+1\right)}{\frac{{\overset{-}{r}}_{r}}{\mu }-{\overset{-}{l}}_{r}\left(\frac{\eta }{\mu }+1\right)} \end{array}$$

where, ${\overset{-}{l}}_{p}$ is the average length of a protein in base pairs, ${\overset{-}{r}}_{r}$ (bp s^−1^) is the maximum base pair processing rate of the ribosome which is assumed to be constant across cell size within bacteria where the ribosomal structure is highly conserved (Petrov et al., 2014), *l*_*r*_ is the average length of a ribosome transcript in bases, and η (s^−1^) and ϕ (s^−1^) are specific degradation rates for ribosomes and proteins respectively. From Equation (4), the mRNA and tRNA requirements, under the assumption of constant local concentration, are given by

(5) $$\begin{array}{l} N_{tRNA}={\overset{-}{n}}_{tRNA}N_{r}, \end{array}$$

and

(6) $$\begin{array}{l} N_{mRNA}={\overset{-}{n}}_{mRNA}N_{r} \end{array}$$

where ${\overset{-}{n}}_{tRNA}$ is the average number of tRNA per ribosome (9.3 Bremer and Dennis, 1996), ${\overset{-}{n}}_{mRNA}$ is the average number of mRNA per ribosome in the cell which has been calculated as 1.08 (Kempes et al., 2016), and *N*_*p*_ is the total number of proteins as described above. In the above equations it should also be noted that the specific growth rate, μ, can be easily connected to overall cell size (Kempes et al., 2012, 2016) which we employ in our calculations.

**Figure 3:**
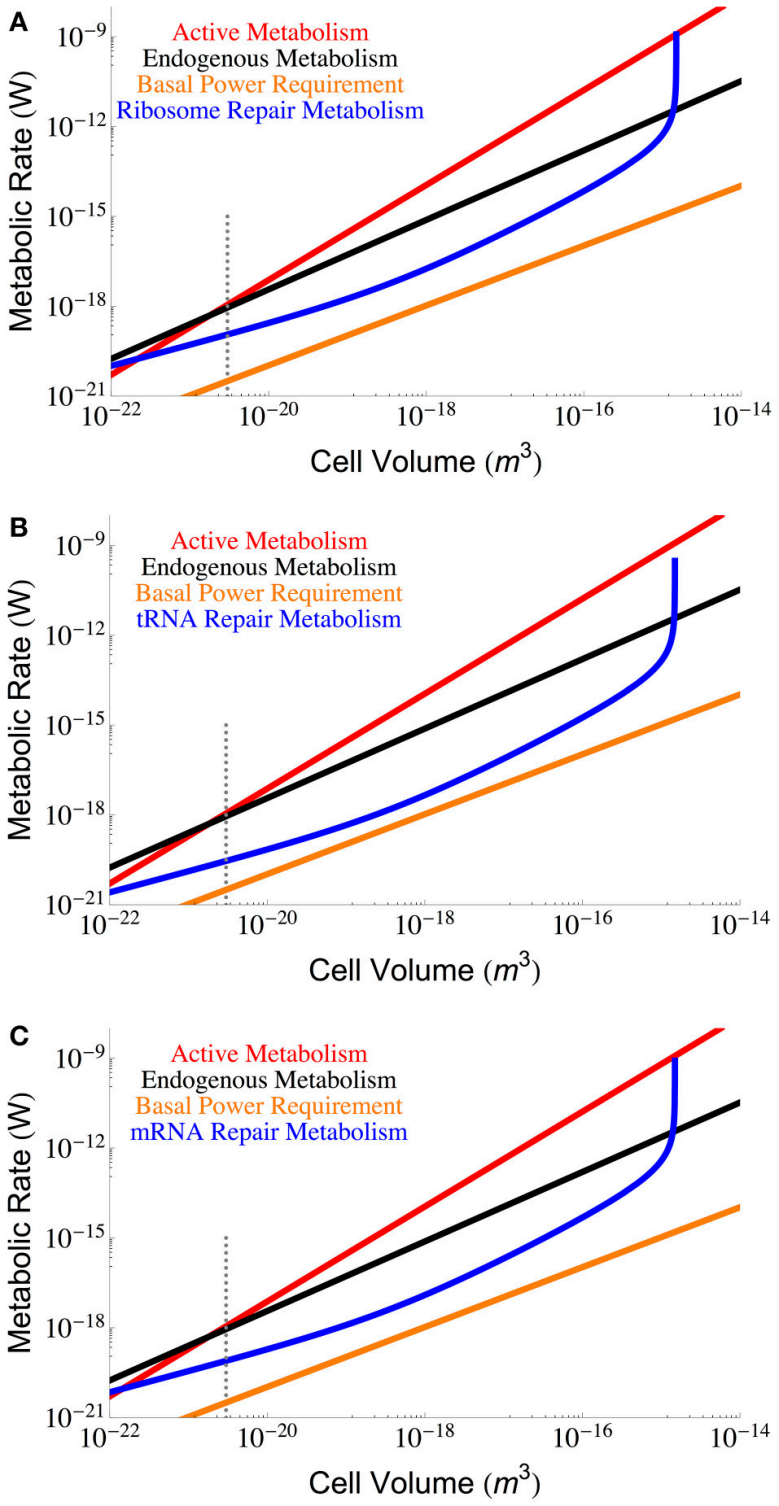
**The overall scaling of total (red) and maintenance (black) metabolism along with predictions for repair cost of (A)** ribosomes, **(B)** tRNA, and **(C)** mRNA. In an attempt to capture less active rates of repair we used the degradation rates reported in Defoiche et al. (2009), combined with reported polymerization costs (Neidhardt and Ingraham, 1990; Russell and Cook, 1995; Pramanik and Keasling, 1997; Haynie, 2001; Lever et al., 2015; Lynch and Marinov, 2015; see Methods Section for a longer discussion). The dotted gray line indicates the smallest known bacterial cells (Luef et al., 2015; Kempes et al., 2016).

Figure 3 gives the maintenance cost for each of the RNA components (tRNA, mRNA, and ribosomal) using the previously described dependence of total RNA abundance on cell size (Kempes et al., 2016), and the measured η_*x*_, and β_*x*_ for each component. Notably, each of the RNA costs scales in the same way as total maintenance metabolism with respect to cell size for most of the range, implying that their percentage cost is approximately invariant in this range. In addition, for small cells the RNA maintenance costs are relatively small compared to the costs associated with proteins, but for the largest cells this cost becomes asymptotically large as cells reach the previously reported “ribosome catastrophe” (Kempes et al., 2016). It should be noted that, with the exception of ribosomes, the RNA repair costs greatly exceed the BPR. Again our calculation does not consider the reduction in RNA concentrations which, similar to proteins, decrease drastically (as much as 65%) under starvation conditions. Similarly, the rates of degradation are also likely to decrease under lower metabolic activity.

### 3.3. The cost of proton gradients

**Figure 4:**
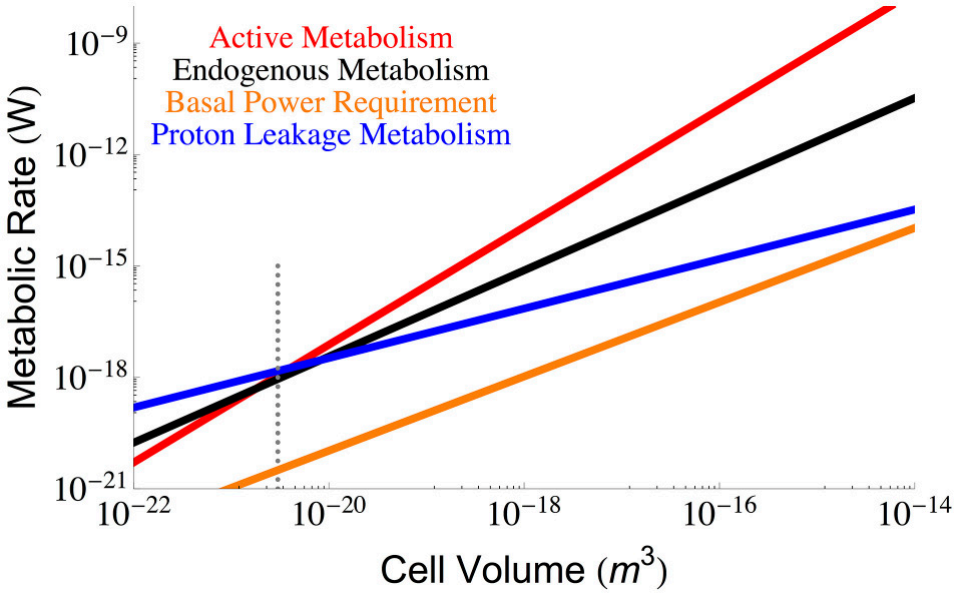
**The overall scaling of total (red) and maintenance (black) metabolism along with predictions for the total maintenance energy due to proton leakage**. Bulk proton leakage rates are from Pramanik and Keasling (1997). The dotted gray line indicates the smallest known bacterial cells (Luef et al., 2015; Kempes et al., 2016).

Beyond the repair of damaged cellular components we must also consider the maintenance of cellular gradients compared with the outside environment (van Bodegom, 2007; Hoehler and Jørgensen, 2013; Lever et al., 2015). From this perspective, a key component of maintenance metabolism is the leakage of ions through the membrane that are not used for ATP synthesis, which is an effective loss of energy as this ion gradient was generated as part of the overall metabolism. This leakage could reflect the overall surface area of the cell, or be dependent on the total number of ATP synthases, transporters, or membrane bound proteins– all of which could change with cell size. We use bulk measurements for proton leakage rates per surface area to calculate the effective ATP, and thus energy, lost per unit surface area, which we then scale to the total surface area of the cell:

(7) $$\begin{array}{l} B_{proton}=l_{proton}6^{2/3}\pi^{1/3}V^{2/3} \end{array}$$

where *l*_*proton*_ is the lost proton power per unit surface area (see Section Methods), and our calculation is presented in Figure 4 as a function of cell volume.

### 3.4. The cost of motility

**Figure 5:**
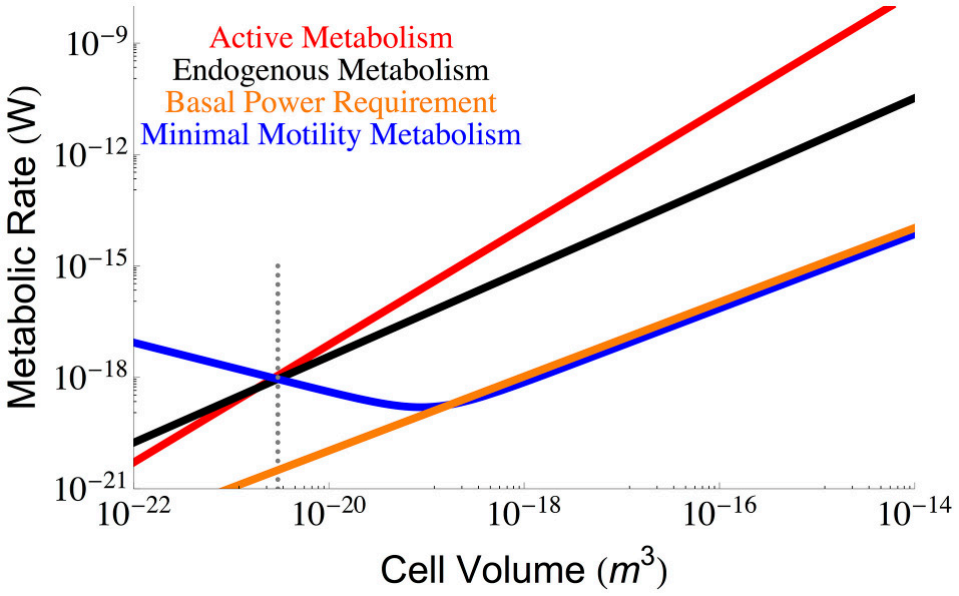
**The overall scaling of total (red) and maintenance (black) metabolism along with predictions for the minimal energy required for cellular motility assuming run-tumble chemotaxis (Berg and Turner, 1990; Mitchell, 2002)**. The dotted gray line indicates the smallest known bacterial cells (Luef et al., 2015; Kempes et al., 2016).

Other considerations of maintenance metabolism have pointed to motility as an important component (van Bodegom, 2007; Hoehler and Jørgensen, 2013; Lever et al., 2015). However, it has been shown that motility is likely to be rare under energy limiting conditions (Biddle et al., 2008; Lever et al., 2015) and that motility is virtually absent in species with cell sizes below 2.68 × 10^−19^ m^3^ (Dusenbery, 1997) (roughly two orders of magnitude larger than the smallest cells), and thus it is unlikely that motility will enter into the BPR. Similarly, because motility has a complicated relationship to energetic return it may not be necessary to count the entire process as part of maintenance (Lever et al., 2015), especially since the repair of motility has potentially already been accounted for in our assessment of protein degradation. For completeness we consider the energetic cost of motility relative to the metabolic rates previously considered. Previous work gives the minimal expenditure on motility as $B_{mot}=\frac{kTD}{r^{2}}+3r^{3}$ (Mitchell, 2002) for a spherical cell, where *k* is Boltzman's constant, *T* is temperature, *D*_*m*_ is the translational molecular diffusion (5.19 × 10^(−6)^ (cm^2^ s^−1^) at 25° Berg and Turner, 1990), and *r* is the cellular radius. We compare this prediction with metabolic rates in Figure 5 where we find that the total motility metabolism is close to and follows the scaling of the BPR, and would only become significant compared with the maintenance and active metabolism for the smallest cells. This result reinforces the idea that motility should not be employed at the smallest scales. Even for large cells, the minimal motility costs are comparable with estimates of BPR, suggesting that motility is unlikely under energy limiting conditions. However, recent metatranscriptomic studies have shown an increased expression of motility genes correlated with porosity (Orsi et al., 2013), which suggests that the employment of motility costs is likely a complicated function of expected return on expenditure. It is thus unclear when precisely these costs would be eliminated in contexts where energy availability is scarce but also where search increases the flux of these energy resources. Certainly there is an energy availability where motility does not yield a positive return and this would be the case in environments where the BPR roughly matches the motility costs (Figure 5).

### 3.5. Fractional and total repair costs

**Figure 6:**
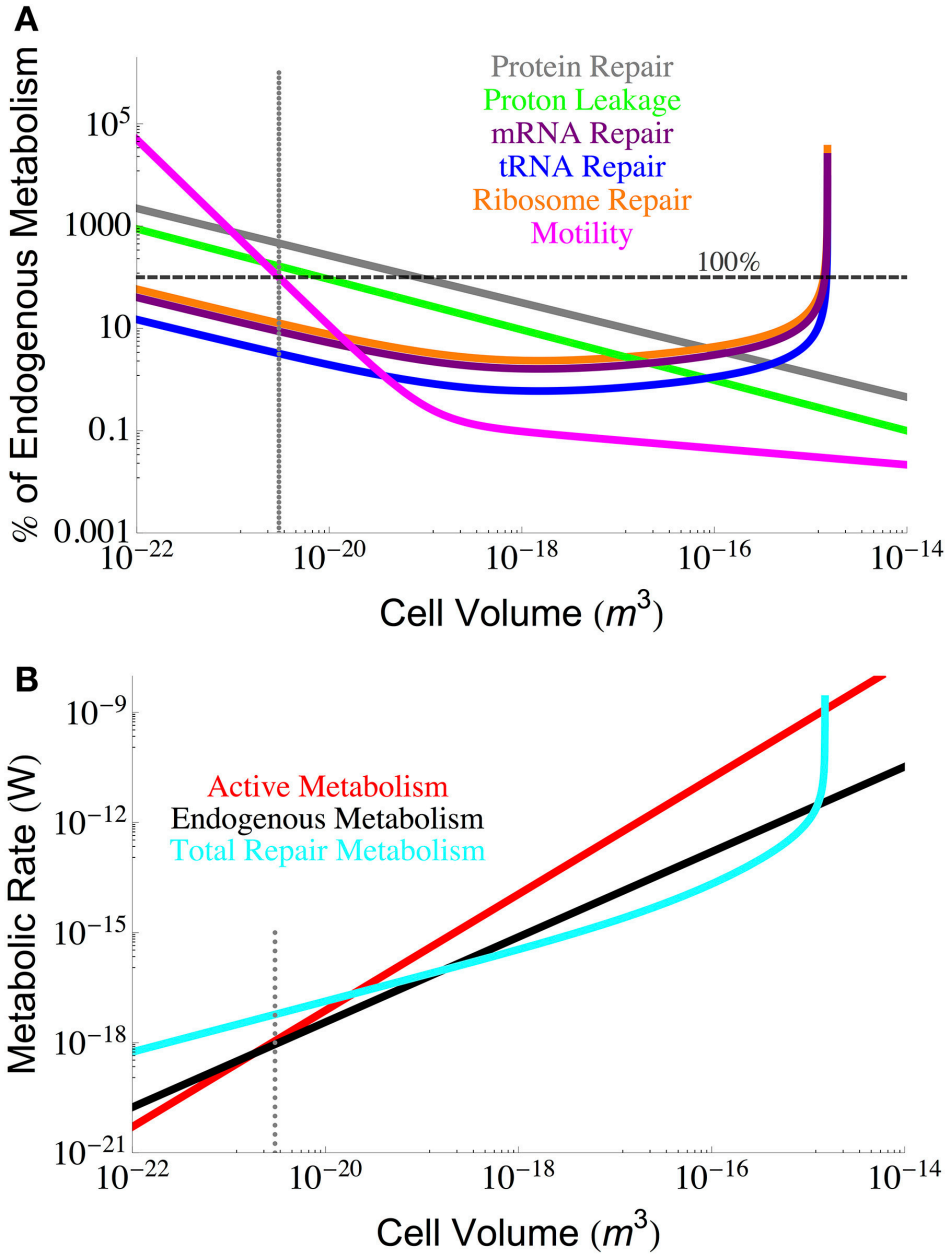
**(A)** Each component of cellular maintenance plotted as a percentage of the measured endogenous metabolism. The horizontal dashed line indicates 100% of the endogenous metabolism, and values which fall above this line are shown as extrapolations of the derived interspecific trends. **(B)** The overall scaling of total (red) and maintenance (black) metabolism along with predictions for the total repair cost (cyan) as calculated from a summation of all individual component repair costs (excluding motility). The total repair costs closely follow the measured endogenous metabolic rates. The dotted gray line indicates the smallest known bacterial cells (Luef et al., 2015; Kempes et al., 2016).

From our analyses it is possible to compare the relative costs of each component of maintenance metabolism. Figure 6 makes this comparison by expressing each component cost as a fraction of the known endogenous metabolic rate, each of which is a function of cell volume. Here we observe that the smallest cells are more constrained by protein repair, motility, and proton leakage, while the largest cells are constrained by RNA components. It should be noted that the protein repair costs are the highest costs for all but the largest cells, and interestingly, the RNA components reach a minimal cost for intermediate cell sizes and only exceed protein costs when the demand for ribosomes becomes asymptotic.

It is also possible to estimate the total maintenance costs by summing the component costs. Figure 6 provides the total maintenance costs as a function of cell size compared to the active and endogenous metabolic rates. In this plot we have excluded motility because of its variation across species and because it can be eliminated as a cost. Remarkably, we find that the total repair metabolism closely follows the curve for endogenous metabolism despite the addition of cost relationships that all have distinct scaling relationships with cellular volume. At the large end of bacteria, total costs become asymptotic at the size where the RNA components are predicted to overwhelm cell volume (Kempes et al., 2016). At the small end, the calculated repair costs exceed both the endogenous and active metabolic rates owing almost entirely to the high cost of protein repair which we have already discussed above.

## 4. Discussion

Our results suggest that at the lowest cell sizes and metabolic rates the cost of maintenance is significant and largely dominated by the cost of repairing proteins. Thus small cells, or cells in energy limiting environments, may be under selective pressures to utilize long-lived proteins, or evolve pathways that are unlikely to either damage or rely on the intentional degradation of proteins as part of regulation. The high relative cost of protein repair agrees with measurements that show that sulfate reducing bacteria and fungi living under extreme energy limitation have elevated expression of protein turnover and chaperone genes (Orsi et al., 2015, 2016). These metatranscriptomic data verify that at low metabolic rates a large fraction of the cell's activity is devoted to protein cycling. DNA repair genes also have a high relative expression, but not to the same degree (Orsi et al., 2015, 2016).

Our results are consistent with the expectations and measurements reviewed in (Lever et al., 2015), but add to this the important consideration of how protein repair processes change across the range of bacterial sizes. For example, larger cells face a much lower cost of repairing proteins relative to any measure of metabolic rate including BPR. This is also true for proton leakage as dependent on membrane surface area. Other processes, namely RNA repair and motility, have a minimum relative cost (compared with cell size or metabolic rates) for intermediate cell sizes. Taken together this set of interspecific trends may imply that there are intermediate cell sizes where maintenance costs are minimized. These intermediate sizes are likely smaller than cells growing under the best conditions, but are significantly larger than the smallest bacteria.

In many of our calculations of constants we had to make assumptions about the cell sizes that corresponded to certain measurements. Given the considerable change in cellular composition across the range of bacteria and the importance of cell size, or size change as a response to poor environmental conditions (Lever et al., 2015), it will be important for future studies to measure an array of cellular features, including cell size, simultaneously and under extreme energy reduction (van Bodegom, 2007; Hoehler and Jørgensen, 2013; Lever et al., 2015) and to do so for a range of bacterial sizes. Our efforts here lay out a framework for understanding the size-based maintenance metabolism across bacteria as connected with the current understanding of cross-species physiological tradeoffs. Higher-order questions about covariation in unit costs, distributions of protein types, or other species-level variations could be considered, along with variation in scaling relationships at more detailed taxonomic levels. Our perspective allows for the incorporation of variation in scaling relationships or constants into the calculations that we have outlined. Future measurements combined with this framework could help to further elucidate the hard constraints, and variation therein, connected with the basal power requirement, and help us access whether deep sediment cells are living and evolving or simply dying slowly (Schippers et al., 2005; Lever et al., 2015).

## 5. Methods

### 5.1. Data compilation

Some of the data compiled in previous studies, which have been summarized here, relied on different modes of conversion. For example, DeLong (2010) and Makarieva et al. (2005, 2008) relied on a constant density conversion from cell volumes to dry weight (used to convert mass specific consumption rates to total rates) while Lynch and Marinov used the known scaling between cell volume and dry weight which has also been employed in other studies (Kempes et al., 2012, 2016). Conversely, DeLong (2010) employ a more mechanistically based temperature normalization involving a Boltzman factor of *e*^−*E*/*kT*^ (where *E* = 0.6 eV is the activation energy; Gillooly et al., 2001) compared with the Q10 methods of (Lynch and Marinov, 2015). We used the stated conversions of each reference to go back to the original mass-specific consumption rates and cell volumes from which we applied the Boltzman factor to both total and maintenance metabolic rates, and uniformly converted volume to dry mass following

(8) $$\begin{array}{l} DW=v_{0}V^{\alpha_{c}} \end{array}$$

where *v*_0_ = 265.46 (dry fg μ${\text{m}}^{-\alpha_{c}}$) and α_*c*_ = 0.89 represent average values for combined scaling relationships from several studies (Norland and Heldal, 1987; Loferer-Krossbacher et al., 1998; Kempes et al., 2012, 2016). Note that the volume to dry weight conversion represents an average of several within and across species scalings opposed to the *E. coli* value used by Lynch and Marinov (2015). In finding the metabolic rates we employed the common conversions of 20 (J·ml O_2_^−1^) (Makarieva et al., 2008, 2005) and 47.7 (kJ mol^−1^ ATP) (Tran and Unden, 1998), and for effective glucose energetics we used the ratio of 3.07 (glucose/O_2_) which is an average from a previous compilation (Heijnen and Roels, 1981), and this number accounts for the effective energetic stoichiometry of glucose given multiple uses in the cell. In addition, metabolic rates were estimated from the growth totals (ATP/cell) by dividing by the generation time for maximum growth, both of which are provided by Lynch and Marinov (2015).

The application of OLS fits in contrast to the previous RMA (reduced major axes regression) results of DeLong (2010) was employed in this paper to facilitate comparisons with past estimations of a variety of data where the field standard has typically been OLS methods (e.g., Brown et al., 2004). In addition, it is often of interest in the literature to combine a variety of scaling relationships to predict others, and if the empirical relationships are a mix of both OLS and RMA fits, then this complicates the interpretation of the algebraic manipulations. However, for future use and flexibility the RMA exponent fits for total metabolism are 1.86 ± 0.24 for our reanalysis of DeLong (2010) and 2.01 ± 0.27 adding the Lynch and Marinov data (Lynch and Marinov, 2015). Similarly, the RMA estimate for the maintenance metabolism exponent is 1.64 ± 0.33.

### 5.2. Constants and calculations

Here we have applied the power-law approximation for growth rate and it should be noted that the more complicated theoretical form of Kempes et al. (2012) (see below) could also be used in various equations, in which case there would be an asymptotic limit for the smallest cells. Similarly, a more detailed perspective of the dynamic rates of a cell at various stages of the division trajectory could also be found using various equations from Kempes et al. (2016).

For the basal power requirement we used the lower bound on metabolic rates of 3.17 × 10^−20^ (W/cell) and a typical cell size of 3 × 10^−20^ (m^−3^), both reported in Lever et al. (2015), to estimate the metabolic rate per unit volume as ζ = 1.06 (W m^−3^). The proton leakage rate has been previously reported as 1.05 × 10^16^ (H^+^ · g DW^−1^ · s^−1^) which can be converted to a leakage per unit volume using Equation (8) and an intermediate cell size (≈ 10^−18^ (m^3^)) corresponding to a dry weight density of 2.65 × 10^5^ (g DW · m^−3^)). We find a per unit volume rate of 2.78 × 10^2^ (H^+^ · m^−3^ · s^−1^) which, for the same cell size, corresponds to a per unit surface area rate of 5.75 × 10^14^ (H^+^ · m^−2^ · s^−1^). Given that 3.1 H^+^ yields one ATP (Tran and Unden, 1998), and using the free energy of ATP, gives an effective power loss of $l_{proton}=1.48\times 10^{-5}$ (W m^−2^).

For the cost of replacing a protein we have that the average protein length is 924 bp or 308 amino acids across across a variety of bacteria (Xu et al., 2006), and we use this in combination with the free energy of ATP and the fact that it requires 4 ATP to polymerize one amino acid (e.g., Lever et al., 2015; Lynch and Marinov, 2015) to calculate $\beta_{p}=9.79\times 10^{-17}$ (J protein^−1^). This cost is combined with the reported half-lives discussed earlier (Collins and Gernaey-Child, 2001; Maier et al., 2011) to calculate the repair power for proteins. Here we are ignoring the cost of returning damaged proteins back into the amino acid pool, which could cost between 0.25 and 1.0 ATP per amino acid (Lynch and Marinov, 2015), because it is unclear exactly what these values would be in bacteria.

For the repair of RNA components we apply a similar strategy of combining half-lives with the polymerization cost per component unit (ribonucleotides in this case) along with the average length of the entire component. For mRNA, reported average half-lives in bacteria range between roughly 5 and 25 min (Bernstein et al., 2002; Hambraeus et al., 2003; Dressaire et al., 2013; Lynch and Marinov, 2015). However, as discussed with the protein decay rates, many of these degradations are likely part of the intentional regulation of cellular physiology, as evidenced by the increase in average half-lives with decreasing cellular growth rate (Dressaire et al., 2013). For this reason we use the much longer half-lives of 7–10 h (Yang et al., 2003; Defoiche et al., 2009) found in mammalian systems which may represent a more natural baseline decay rate and more appropriately correspond to endogenous metabolism. Similarly, for the decay of ribosomes and tRNA we use the half-lives from Defoiche et al. (2009), which are, respectively, 7.0 and 0.6 days (these values are comparable to the range of 65–79 h for rRNA; Nwagwu and Nana, 1980 and 44–72 h for tRNA Phizicky and Hopper, 2010). The cost of replacement for mRNA, β_*mRNA*_, is found by considering 1 ATP per polymerization (the commonly used range is 0.3–2 Neidhardt and Ingraham, 1990; Russell and Cook, 1995; Pramanik and Keasling, 1997; Haynie, 2001; Lever et al., 2015; Lynch and Marinov, 2015) and the length of the average gene given above. The cost of replacement for a ribosome, β_*ribosome*_, is given by the cost of remaking both the rRNA and the ribosomal proteins, where there are 4566 ribonucleotides and 7336 amino acids per ribosome (Bremer and Dennis, 1996) and the respective polymerization energiers are described above. Likewise, β_*tRNA*_ is found by considering that the average length of a tRNA is 80 nucleotides (Bremer and Dennis, 1996) and again using the above RNA polymerization ATP requirements.

It has been demonstrated (Kempes et al., 2012) that the metabolic scaling relationship for active and endogenous metabolism can be used to derive growth rate, μ (s^−1^), following the relationship

(9) $$\begin{array}{l} \mu =\frac{\left(B_{m}/E_{m}\right)\left(1-\alpha_{B}\right)\ln\left[\epsilon \right]}{\ln\left[\frac{1-\left(B_{m}/B_{0}\right){\left(V_{c}d_{c}\right)}^{1-\alpha_{B}}}{1-\epsilon^{1-\alpha_{B}}\left(B_{m}/B_{0}\right){\left(V_{c}d_{c}\right)}^{1-\alpha_{B}}}\right]} \end{array}$$

where α_*B*_ and *B*_0_ are, respectively, the scaling exponent and normalization constant for active metabolism, *d*_*c*_ (g m^−3^) is cell density, ϵ ≈ 2 is the ratio of the size of the cell at division compared with its initial size, *B*_*m*_ (W g^−1^) is unit maintenance metabolism, and *E*_*m*_ (J g^−1^) is the unit cost of biosynthesis (see Kempes et al., 2012 for details on how these last two parameters can be derived from bulk community energetic constants).

## Author contributions

CK and PMvB conceived of the study. CK carried out the initial theory development and analysis. All authors contributed to the development of the theory, discussed the results and implications, and contributed to writing the manuscript.

## Funding

CK acknowledges the support of the Omidyar Fellowship at the Santa Fe Institute, the Life Underground NASA Astrobiology Institute (NNA13AA92A), the NASA Exobiology Program (NNX16AJ59G), and the Gordon and Betty Moore Foundation. EL thanks the Omidyar Fellowship at the Santa Fe Institute for support.

### Conflict of interest statement

The authors declare that the research was conducted in the absence of any commercial or financial relationships that could be construed as a potential conflict of interest.
